# Graduate Study on the Continent—Russia

**Published:** 1909-03-27

**Authors:** 


### GRADUATE STUDY ON THE CONTINENT—RUSSIA.

### I. SOME PRELIMINARY REMARKS.

We were told, confidently and authoritatively, before we left England that a visit to Russia would furnish relatively little of importance from a medical graduate's point of view. Russia, we were told, lies in the twilight of the medical history of yesterday. It was not expressed so metaphorically, it is true, but that, in fine, was the abstract of our information.

Let us say at once that the man who travels to Russia in the belief that he will be able to add to his collection of prehistoric surgical instruments or witness antiquated methods of treatment, who goes thither to find some laudable delight in contrasting the advance in his own country with the backwardness of the country whose guest for the time being he is, will be wholly disappointed. There are old instruments, but they are treasured in glass-cases, and among them he will be scandalised to find some with which he familiarised himself as a dresser, and which he, mayhap, still reverently cherishes. There are antiquated methods of treatment, but they linger in the small towns, just as in Devonshire spider's web is still regarded as the best hæmostatic. What the graduate will find is a splendid system of hospitals, none of them palatial or comparable to the best German or American institutions (with the exception, perhaps, of the new Child Jesus Hospital at Warsaw), but all serviceable, hygienic, well equipped, and, to a great extent, admirably managed. Especially will he find a praiseworthy extension of the small hospital system. Every large town in Russia possesses one or more hospitals, giving a proportion of bed space to the total population which is not far behind that which obtains in other countries. What is more striking, the majority of small towns and villages possess a cottage hospital. This is often an undignified and not very up-to-date building, but it is there, and it does good work, while many of the newer hospitals in the smaller towns are really models of their kind.

This, perhaps, is the first thing that impresses the visitor, and possibly it gives him an erroneous idea of the advance of medicine in Russia. For just as absurd as it is to underrate the advance of the country in matters of hygiene and hospital construction, so it is equally absurd to overestimate the progress that has been made. A great deal remains to be done. The population is so large, and disease, the harvest of long neglect, bad education or the want of education, overcrowding, and neglect is so rampant that there is room and material for double the number of existing hospitals. Nevertheless, the visitor is impressed with the progress that has been made, and a visit to a few Russian hospitals will greatly augment his respect for his Russian colleagues and his admiration for their methods.

At the outset it may be remarked that Russia, in the matter of post-graduate study in its strictest sense, holds out very few attractions to the English speaking practitioner. Travelling is difficult and expensive; the language, one of the most stubborn for a Teutonic tongue to master, is a great obstacle to the perfect enjoyment of a visit to the country: correspondence and books present other difficulties, and, to sum up, the peculiar customs which prevail at the various hospitals are often matters of great inconvenience, not to say of some annoyance. To take the first difficulty, no graduate should presume to travel in Russia without a proper passport, *viséd* by a Russian Consul at the city of residence of the graduate, and *revisèd*, for safety's sake, at every place where he has made a somewhat prolonged stay. This passport is in constant requisition, but we may say that Russian officials are not more fearsome beings than the ordinary Prussian policeman. Here, as elsewhere, the graduate who comes to the Continent for purposes of study should be prepared to answer impertinent questioning with the diplomatic discretion of a Talleyrand. On arrival at the frontier the passport is requisitioned by the officials, and the traveller has to wait in his carriage until it is returned to him—often a matter of considerable delay, especially when he travels in an ordinary instead of an express train. On arrival at a town the passport is handed to the hotel-keeper, who usually arranges all police matters for his guests. The graduate who wishes to make a prolonged stay in one place, or even to remain for a while in the country, visiting several towns in succession, will find things by no means so easy. He will have to go to the police office, where, after prolonged parleying, he can obtain, if his credentials are entirely satisfactory, a sojourn permit which allows him to live for a month within the domains of the great White Czar. Such a permit is an essential preliminary to renting rooms or staying at a pension, neither of which we recommend to a graduate who is not able to fight his way along with a thorough knowledge of the language. At the end of a month, "if his behaviour has been such as to justify his privilege being granted by the authorities," he may obtain an extension of this permit for one year. Both permits, it may be mentioned, are subject to summary withdrawal. On leaving the country the passport is again demanded at the frontier, and, if the traveller has stayed more than a month in the country, special permission to leave must be written on it, and it must be stamped by the local police bureau. For a fleeting visit this latter proviso is not necessary, and usually no difficulty is experienced at a frontier station if the passport is in order. Railway travelling is comparatively expensive, unless the student travels third class, a method which we strongly advise him not to adopt, as it is uncomfortable in the highest degree, and adds greatly to the annoyances he will experience in his travels. The second and first class compartments, on the contrary, are comfortable and good, and the fellow travellers one meets are usually able to speak another language besides Russian, and in many ways to help the stranger. Even more expensive is travelling by carriage or cab. The drivers always overcharge, and the Russian always bargains with his cabman. The stranger, therefore, is at a great disadvantage, and must content himself with the excellent electric tramcars which run in most of the larger towns, and in which the fares are generally low.

Coming to the second difficulty, the language, it may be stated that for a short sojourn the stranger would do well not to attempt to master the intricacies of a tongue that is really a combination of three systems, and unusually difficult for a foreigner to master. At the most he will learn a few catch phrases and general terms—such as the absurd method of calling the waiter "You person you," the chambermaid "You she-individual," and so on. At the large hotels—which are relatively more expensive, but perhaps more comfortable, than similar ones in Germany—he will find that a knowledge of German, French, or English is sufficient. For all practical purposes a knowledge of German will be of most use to him. Nearly all the Professors, and many of the practitioners, speak it, although lectures in German are given at only one Russian University, that of Dorpat. Many professional men speak English fluently and well, while others again are proficient in French. The language difficulty will, of course, be chiefly encountered in the out-patient department, and in getting about in the smaller towns. Thus with the porters at the large hospitals the stranger will often have to make himself understood by sign language, or, better, by that *lingua franca* which is common to all mankind—the language of the discreet and serviceable tip—and his best etymological *vade-mecum* will be a pocketful of five kopeck bits.

The third difficulty will be with books. At the frontier the traveller will find his library the object of the closest interest, and it is no easy matter for one unacquainted with Russian to explain to the Customs official that an extract from the "Annals of Surgery," containing a photograph of Dr. Samuel Robinson's high-pressure lung cabinet, is not a delineation of a new chemical apparatus for manufacturing explosives. The graduate would do well, therefore, to leave his professional books, except such as are obviously scientific, behind when he visits Russia. Let us say again that Russian officials are no more discourteous or unfriendly than German ones. They have a system of rules and must keep to them, and it is hardly fair to expect them to make more allowances for an English graduate than for any other individual. Courtesy, a frank declaration of one's effects, and a little blindness to the domineering, militant manner which is a characteristic of all officialdom, and which sometimes puts up the back of the freeborn, are all that are required, and they usually bring the traveller safely through all Custom houses and across all frontiers.

Finally, we come to hospital customs, and here the unwary may sometimes find a source of annoyance. To begin with the hospital porter, generally a huge being dressed like a Lord Chancellor and as important as a Lord Mayor, guards the hospital entrance jealously, and it is sometimes not a little difficult to get past this individual. Then again there are many festival days on which visits to the hospital are not looked upon with favour. Such days are holidays for the staff and patients alike, and the porter is dressed in an even more imposing costume. In the wards the visitor will find himself more or less at home, for there is little difference between the inside of a Russian hospital (if one excepts religious decorations and the pictures of the Czar) and that of a well-managed English institution. The diagnosis of each case is writ large, usually in Latin, on the blackboard over each bed, and the physician in charge of the ward is always most courteous, and willing to show the visiting colleague round. At this stage, then, we may qualify our original observation and add that Russia, in the matter of graduate study in its widest sense, holds out many attractions. That is to say, to the graduate who travels abroad not merely to attend courses, but to enlarge his whole purview, a visit to the country and an inspection of its hospitals cannot fail to be of interest and to be fraught with instruction and enjoyment. In a future article we shall deal more fully with graduate study in its strict sense in Russia—here we may remark in passing that such study is not for the man who does not know Russian well—and go on to consider what the mere visitor, interested in medical matters, may learn from a brief visit to the country.

We have already pointed out that Russia possesses many hospitals, and that these, without exception, are well worth seeing. The Warsaw institutions are new, up-to-date, and full of interesting, and, in some ways, out-of-the-way cases. Among the chief are the large Child Jesus Hospital and the equally fine military lazaret. At Moscow there are several fine hospitals, and at St. Petersburg the University clinics are among the best in Europe. The Professors at the St. Petersburg are well-known as specialists in their various subjects—we need only here mention the names of Professor Pawlow, of the Imperial Physiological Institute, and of Professor Bechterew, of the Imperial University Clinic for Psychiatry and Neurology. The operating theatres are usually fine and modern, and the work done in them is in many ways pioneering. Thus, to mention one fact only, at Warsaw we found the surgeons interested in the transplantation of joints, and a series of cases were to be seen in which these interesting experiments had been carried out with excellent results. In pure medicine the student will find a large number of subjects of great interest in Russian hospitals. The clinical material for the study of fevers especially is unusually large and rich, and nervous diseases, diseases of the eye, of the intestinal tract, and of the lungs are well represented.

Going round the wards with the physicians or attending operations with the surgeon and examining out-patients is the most that strangers can do. Clinical lectures, which are regularly given, are, of course, in Russian or Polish, and useless to the graduate who does not know either language. Attendance at out-patients, for the same reason, has its drawbacks, but it has this advantage that the student will learn to appreciate the importance of correct observation and examination without reference to history.

Finally the graduate will find it exceedingly useful to come to Russia provided with introductions to the colleagues he is to meet. Such introductions will largely contribute to the enjoyment he will derive from his visit. The Russians are a hospitable race, and the medical profession in Russia is not behindhand in showing a colleague every courtesy and kindness, often at personal sacrifice to themselves. The graduate visitor will find much to interest him, not the least in the new conditions of life around him, and will return from his Russian trip with a greatly increased respect for the professional standing of his colleagues in Russia, and with a better knowledge of that interesting country which will be of use to him in after life.

